# Whole-genome DNA methylation analysis of Chinese hamster ovary cells undergoing media adaptation

**DOI:** 10.3389/fbioe.2026.1716758

**Published:** 2026-01-21

**Authors:** Suki Roy, Jasrene Kaur Sandhu, Lingzhi Huang, Abraham Wong, Guo Xuan Wan, Frank Lyko, Emeka Ignatius Igwe, Florian Böhl

**Affiliations:** 1 Evonik (SEA) Pte Ltd, Asia Research Hub, Singapore, Singapore; 2 Division of Epigenetics, DKFZ-ZMBH Alliance, German Cancer Research Center, Heidelberg, Germany; 3 Creavis, Evonik Operations GmbH, Hanau, Germany

**Keywords:** Chinese hamster ovary (CHO) cells, DNA methylation, epigenetics, media adaptation, whole-genome bisulfite sequencing (WGBS), DNA methylation array

## Abstract

**Introduction:**

Chinese Hamster Ovary (CHO) cells are widely used for the production of recombinant therapeutics due to their ability to carry out human-like post-translational modifications. Media adaptation represents a key step in large-scale production to ensure optimal safety and cost efficiency. As DNA methylation is a central epigenetic mechanism underlying adaptive modulation of gene expression, we report here, for the first time, the use of high-coverage whole-genome bisulfite sequencing to generate single-base-resolution maps of CHO cells at different phases of growth in a fed-batch culture and undergoing media adaptation across four different media.

**Methods:**

A CHO cell line was adapted to four commercially available media, and their growth rates and productivity were compared with those obtained using the control medium in a 7-day batch culture. This approach resulted in the generation of *n* = 57 high-quality whole-genome DNA methylation datasets, which were subjected to differential DNA methylation and gene association analyses. In addition, we developed a novel DNA methylation array comprising more than 63,000 CpG methylation sites across the CHO genome, enabling streamlined and efficient DNA methylation profiling of CHO cells.

**Results:**

Analysis of *n* = 57 high-quality DNA methylation datasets revealed altered DNA methylation patterns across different phases of growth in a fed-batch culture and in response to distinct media adaptations. Specifically, adaptation to four different media resulted in highly specific methylation changes that were associated with distinct functional outcomes, including protein productivity. Finally, the customized DNA methylation microarray platform was used to validate all media adaptation-dependent epigenetic changes identified by whole-genome bisulfite sequencing (WGBS).

**Conclusion:**

These findings identify and characterize dynamic DNA methylation changes occurring during media adaptation and support their potential use as predictive indicators of CHO cell phenotypic changes in response to a dynamic culture environment. Furthermore, this work represents a valuable resource for the development of DNA methylation-based biomarkers for the optimization of CHO cell culture.

## Introduction

The production of recombinant therapeutic proteins, particularly monoclonal antibodies (mAbs), continues to be a key driver of growth in the biopharmaceutical industry (Dahodwala and Lee, 2019; Walsh and Walsh, 2022). Chinese Hamster Ovary (CHO) cells remain the most frequently used expression system for the production of recombinant therapeutics, primarily due to their ability to catalyze human-compatible post-translational modifications (PTMs) (Dahodwala and Lee, 2019; Marx et al., 2022; Pereira et al., 2018). Additional benefits conferred by CHO cells include their ease of adaptation to serum-free, chemically-defined media, their reduced risk of propagating human viruses, and their amenability to genetic modifications that enable high production levels of recombinant proteins (Dahodwala and Lee, 2019; Kantardjieff and Zhou, 2014; Marx et al., 2022; Walsh and Walsh, 2022; Zeh et al., 2024). Nevertheless, the production of biotherapeutics from CHO cells must be strictly regulated in order to uphold high safety levels for patients (Coulet et al., 2022; Marx et al., 2022).

One of the most crucial parameters required to be maintained for biologics production is the monoclonality of cell lines (Marx et al., 2022). Environmental variations encountered over the course of a fed-batch or batch culture have been found to have significant effects on the performance of CHO cells, driving the emergence of genetically unstable heterogeneous populations with distinct phenotypes, including mAb productivity and quality (Hernandez et al., 2019; Pereira et al., 2018; Yang et al., 2010). This issue is of special concern in the context of biopharmaceutical production, as CHO cells are cultured over long periods of time as part of the industrial scale up process (Feichtinger et al., 2016; Yang et al., 2010). The long culture period allows the accumulation of lactate, ammonia and reactive oxygen species–toxic metabolites that hinder cell growth and affect product quality, and the depletion of glutamine–an essential nitrogen source for protein production in CHO cells (Chitwood et al., 2021; Ha et al., 2022; Pereira et al., 2018; Rajendra et al., 2012). The regular addition of feeds during fed-batch cultures can also contribute to environmental changes in the form of hyperosmolar conditions, which affects cell growth and protein productivity (Alhuthali et al., 2021; Ha et al., 2022; Romanova et al., 2021). Moreover, temperature shifts are commonly used in industrial production settings to promote an earlier transition to the stationary phase, during which protein production increases, but which has also been found to contribute to changes in protein quality (Ha et al., 2022; Xu et al., 2019). As a result, CHO cells frequently face various adaptive pressures that can compromise the monoclonality of cell lines as well as product quality, thereby jeopardising regulatory approval (Hernandez et al., 2019; Marx et al., 2022; Yang et al., 2010).

Recent studies have uncovered a link between phenotypic changes and epigenetic regulation of gene expression in CHO cells in response to a dynamic culture environment (Wippermann et al., 2015; Yang et al., 2010). Specifically, these studies have focused on the connection between gene silencing via DNA methylation and the production instability observed to occur in CHO cells over the period required to scale up these cultures (Hernandez et al., 2019; Wippermann et al., 2015; Yang et al., 2010). CHO cells have also been found to modify their DNA methylation patterns in response to changing levels of available nutrients and metabolic products within a batch culture and in response to adaptation to different media (Feichtinger et al., 2016; Hernandez et al., 2019). Occasionally, this can lead to transgene silencing, resulting in decreased protein production by the cells (Marx et al., 2022; Osterlehner et al., 2011; Yang et al., 2010). Consequently, a better understanding of how CHO cells use DNA methylation to regulate their gene expression is required in order to optimise bioprocessing and enhance the protein productivity of these cells (Marx et al., 2022).

We have previously used whole-genome bisulfite sequencing to establish single-base resolution DNA methylation maps of eight independent CHO cell lines (Böhl et al., 2024). Our results showed that methylation patterns clustered clearly along the three main branches of CHO evolution (Böhl et al., 2024). Here we present evidence of CHO cells altering their DNA methylation patterns in response to changing environmental conditions. We first studied the growth phases of CHO cells, as growth phases affect cell metabolism, nutrient utilization, and protein production in distinct ways. Despite culture conditions remaining relatively constant, we found that CHO cells significantly modify their methylation patterns over the course of a fed-batch culture. Subsequently, we investigated the effects of metabolic stresses and adaptation to different media which is an essential step in bioprocessing to gradually adjust cells to the chemically defined media used in large-scale production (Feichtinger et al., 2016; Nolan and Lee, 2011; Pereira et al., 2018). We identified pronounced differences in genome-wide methylation patterns between adapted and non-adapted cells, with affected genes found to fall within pathways corresponding to phenotypic changes observed in culture. Together, these findings provide support for the use of DNA methylation alterations as a predictive tool to determine changes in CHO cell phenotype that occur as a result of a dynamic culture environment.

## Results

In an initial set of experiments, we analyzed Humira431 cells grown over 14 days in a standard fed-batch culture. The average viable cell density (VCD) of three biological replicates grown in EX-CELL Advanced CHO Fed-batch media over a period of 14 days revealed three distinct phases of growth (Supplementary Figure S1a): the lag phase (days 0–3), the exponential phase (days 4–11) and the stationary phase (days 11–14). We then investigated the effects of various metabolic and cell culture challenges, such as hyperosmolality, lactate accumulation, and no glutamine availability on the cellular phenotype including DNA methylation patterns of the cells. Our cell culture results showed that all the aforementioned metabolic and environmental stresses were able to induce noticeable phenotypic changes by enhancing protein productivity on the final day of culture (Supplementary Figure S1b). However, DNA methylation analysis by whole-genome bisulfite sequencing was unable to identify any significant differences in the DNA methylation patterns between control cells and stressed cells (data not shown).

**FIGURE 1:**
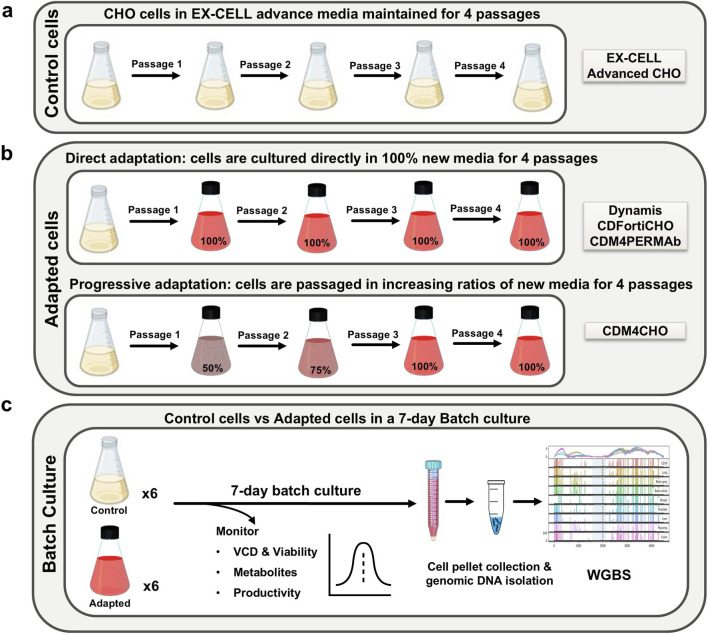
Experimental design for media adaptation in CHO cells. **(a)** Non-adapted Humira431 CHO controls were cultured in EX-CELL Advanced CHO media over four passages concurrently with Humira431 Cultures undergoing media adaptation. Cultures were passaged at mid-exponential phase. **(b)** In the direct adaptation experiments, Humira431 CHO cells were cultured into 100% Dynamis, CDFortiCHO or CDM4PERMAb media. **(c)** in the progressive adaptation experiment, Humira431 CHO cells were cultured into a 50:50 ratio of EX-CELL Advanced CHO and CDM4CHO Media. Subsequent passages increased the ratio of CDM4CHO media to 75% on the second passage and 100% on the third and fourth passage. All cultures were done in six independent flasks for a 7-day batch culture at 37 °C, 8% CO2, and 150 rpm. Viable Cell Density, metabolites and productivity were monitored from day 3 onwards, and cell pellets were collected at the end of the batch culture for genomic DNA isolation.

**FIGURE 2:**
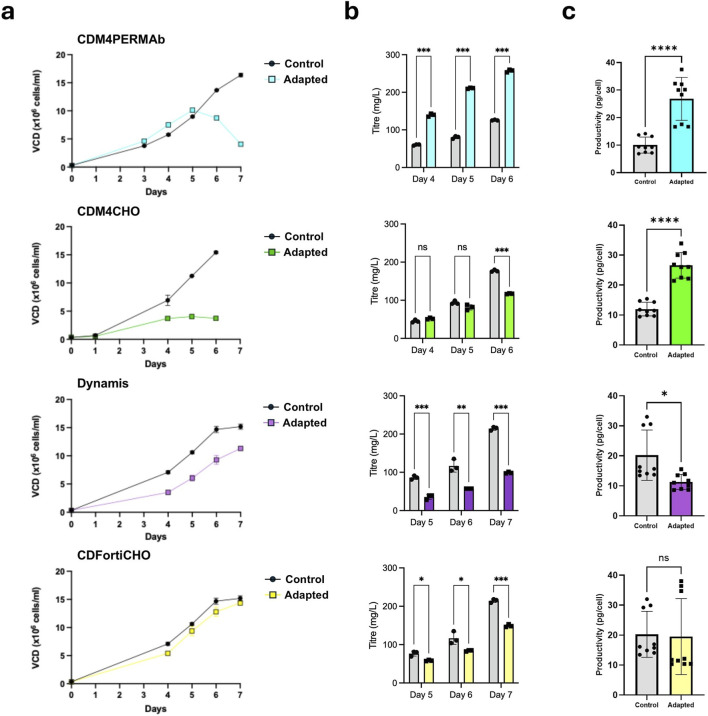
Comparison of growth, protein titre, and productivity of CHO cells grown in batch culture after adaptation to four different media. **(a)** Average viable cell densities of three biological replicates of Humira431 cells measured on different days of growth in the indicated media **(b)** Average titre of three biological replicates of Humira431 cells measured over the last 3 days of culture in the indicated media **(c)** Average productivities from nine biological replicates of Humira431 cells measured on the last day of culture in the indicated media. Asterisks show statistical significance: *p < 0.05, ****p < 0.0001 (t-test).

In a second set of experiments, we used media adaptation to assess the effects of more complex environmental changes on the cellular phenotype including DNA methylation. Therefore, we adapted Humira431 cells growing in EX-CELL Advanced CHO Fed-batch media to four distinct commercially available media (CDM4PERMAb, CDM4CHO, Dynamis and CDFortiCHO) over a period of 2 weeks (Figure 1). Our results showed that cells adapted to CDM4PERMab, CDM4CHO and Dynamis media grew significantly (p < 0.05, Student’s t-test) slower than controls, and no significant difference was observed for CDFortiCHO-adapted cells (Figure 2a). Growth dynamics varied between media with peak VCDs ranging between 3.7 and 14.4 × 10^6^ cells/mL for adapted cells and between 13.7 and 15.4 × 10^6^ cells/mL for cells growing in EX-CELL Advanced CHO Fed-batch media. Parallel analysis of antibody titres and productivity was performed (Figures 2b,c). The antibody titres of Humira431 cell adapted to CDM4CHO, Dynamis and CDFortiCHO media were consistently lower than those of the control groups; whereas the antibody titres of Humira431 cells adapted to CDM4PERMAb were consistently higher than those of the control group, even with declining cell counts toward the end of the growth phase (Figure 2b). Interestingly, Humira431 cells adapted to either CDM4PERMab or CDM4CHO media showed a significant (p < 0.0001, t-test) >2-fold increased protein productivity compared to cells growing in EX-CELL Advanced CHO Fed-batch media (Figure 2c). In contrast, cells adapted to either Dynamis or CDFortiCHO media showed moderately decreased or unchanged productivity on the final day of culture when compared to cells growing in EX-CELL Advanced CHO Fed-batch media (Figure 2c). Altogether, these results demonstrate significant bidirectional changes in productivity between adapted and non-adapted cultures and raise the possibility that changes in DNA methylation might underpin the observed phenotypic differences.

To analyze DNA methylation at the genome scale and at single-base resolution, we used whole-genome bisulfite sequencing (WGBS) (Lister and Ecker, 2009), followed by the identification of differentially methylated regions (DMRs, see Methods for details). In an initial analysis, we used this approach to generate methylation maps from three biological replicates of the three phases of CHO cell growth. In total, we thus generated n = 9 high-quality sequencing datasets, which were characterized by very high yields, high mapping efficiencies and high deamination efficiencies (Table 1). Subsequent DMR analysis found 407 significantly differentially methylated regions between the lag and exponential phases, 1,090 significant DMRs between the exponential and stationary phases and 1,445 significant DMRs between the lag and stationary phases (Supplementary Figure S2a). These DMRs were largely non-overlapping between the three phases (Supplementary Figure S2b), consistent with the notion that they represent dynamic methylation changes during specific phases of growth.

**TABLE 1:** Complete list of WGBS sequencing datasets (n = 57). Altogether fifty-seven whole-genome bisulfite sequencing datasets were generated for the different experiments. The key performance parameters such as number of raw reads, conversion rate, coverage and mapping efficiency are listed.

Experimental group	Culture type	Sample name	Sampling day	No. of raw reads	Mapping efficiency (%)	Conversion rate (%)	Sequencing coverage (x)
Growth curve	Control	HUM FB2-1 D3	Day 3	1,065,680,938	76.9	99.5	63.94
		HUM FB2-2 D3		937,346,880	77.3	99.6	56.24
		HUM FB2-3 D3		784,384,840	75	99.5	47.06
		HUM FB2-1 D7	Day 7	795,194,978	75.9	99.5	47.71
		HUM FB2-2 D7		832,383,280	77.2	99.5	49.94
		HUM FB2-3 D7		860,654,558	77.2	99.5	51.64
		HUM FB2-1 D14	Day 14	924,353,806	64.8	99.5	55.46
		HUM FB2-2 D14		1,033,013,520	72.8	99.6	61.98
		HUM FB2-3 D14		933,708,414	74	99.6	56.02
Dynamis adapted	Control	HM_DYN_D7C1	Day 7	913,237,694	77.1	99.5	54.79
		HM_DYN_D7C2		1,000,711,962	75.5	99.5	60.04
		HM_DYN_D7C3		1,114,688,258	74.1	99.5	66.88
		HM DYN2 D7 C1		1,043,291,312	70.7	99.5	62.60
		HM DYN2 D7 C2		855,115,456	74.7	99.5	51.31
		HM DYN2 D7 C3		809,542,898	72.7	99.5	48.57
	Adapted	HM_DYN_D7A1	Day 7	853,594,254	70.5	99.5	51.22
		HM_DYN_D7A2		836,962,602	74.9	99.6	50.22
		HM_DYN_D7A3		915,544,862	75.6	99.6	54.93
		HM DYN2 D7 A1		771,420,686	73.8	99.5	46.29
		HM DYN2 D7 A2		851,376,844	73.5	99.5	51.08
		HM DYN2 D7 A3		942,427,126	72.3	99.5	56.55
CDM4CHO adapted	Control	HUM CDM4 D6 C1	Day 6	913,408,106	64.1	99.6	54.80
		HUM CDM4 D6 C2		782,966,902	69.2	99.6	46.98
		HUM CDM4 D6 C3		1,167,229,404	71.7	99.6	70.03
		CDM4b D6 C1		844,992,758	76.1	99.6	50.70
		CDM4b D6 C2		901,104,084	76.9	99.5	54.07
		CDM4b D6 C3		1,139,628,728	77.3	99.6	68.38
	Adapted	HUM CDM4 D6 A1	Day 6	917,459,100	73.5	99.5	55.05
		HUM CDM4 D6 A2		780,056,890	72.5	99.6	46.80
		HUM CDM4 D6 A3		786,910,192	72	99.6	47.21
		CDM4b D6 A1		826,817,692	75	99.5	49.61
		CDM4b D6 A2		982,541,566	74.9	99.6	58.95
		CDM4b D6 A3		977,348,898	73.8	99.6	58.64
CDFortiCHO adapted	Control	Hum-MACDF D7 C1	Day 7	926,446,824	72.2	99.5	55.59
		Hum-MACDF D7 C2		825,236,884	72.7	99.5	49.51
		Hum-MACDF D7 C3		995,510,818	72.2	99.5	59.73
		HUM MA2 D7 C1		995,845,242	77.5	99.6	59.75
		HUM MA2 D7 C2		742,873,890	78.1	99.7	44.57
		HUM MA2 D7 C3		988,580,106	77.2	99.6	59.31
	Adapted	Hum-MACDF D7 A1	Day 7	968,894,056	73	99.5	58.13
		Hum-MACDF D7 A2		1,084,010,722	73.1	99.5	65.04
		Hum-MACDF D7 A3		911,331,914	71.6	99.5	54.68
		HUM CDF2 D7A1		819,363,562	77.2	99.6	49.16
		HUM CDF2 D7A2		916,420,164	77.7	99.6	54.99
		HUM CDF2 D7A3		915,329,686	77.4	99.6	54.92
CDM4PERMab adapted	Control	HM Per2 D6 C1	Day 6	1,014,347,474	72.1	99.7	60.86
		HM Per2 D6 C2		986,344,814	77.1	99.6	59.18
		HM Per2 D6 C3		1,151,363,602	73.9	99.6	69.08
		HM PER3 D6 C1		779,018,942	72.4	99.5	46.74
		HM PER3 D6 C2		931,453,126	75.7	99.5	55.89
		HM PER3 D6 C3		758,957,122	71.9	99.5	45.54
	Adapted	HM Per2 D6 A1	Day 6	1,048,606,608	72.6	99.6	62.92
		HM Per2 D6 A2		1,112,631,044	71.5	99.5	66.76
		HM Per2 D6 A3		788,711,822	68.6	99.6	47.32
		HM PER3 D6 A1		748,882,924	71.5	99.5	44.93
		HM PER3 D6 A2		751,743,610	69.5	99.5	45.10
		HM PER3 D6 A3		1,035,694,686	68.2	99.5	62.14

**FIGURE 3:**
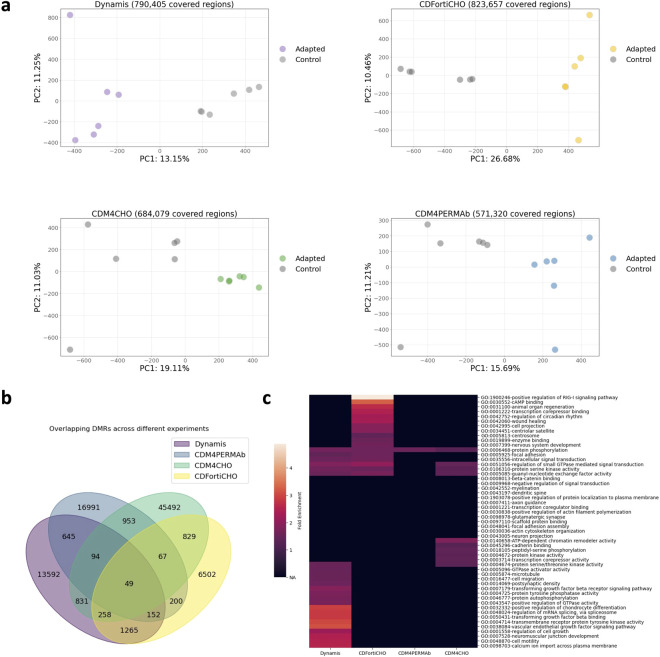
DNA methylation analysis of CHO cells grown in different media in a CHO fed-batch culture. **(a)** Principal component analyses for matched media adaptation and control datasets, based on the beta values of all covered regions. **(b)** Venn diagram showing overlapping differential methylated regions (DMRs) as identified by methylKit. **(c)** Heatmap showing significant GO terms for DMR-associated genes in all four adaptation experiments.

To investigate the methylation changes associated with media adaptation, WGBS analysis was conducted on six biological replicates each that were collected on the final day of batch culture for both adapted and non-adapted CHO cells. This generated n = 48 sequencing datasets of a high quality suitable for downstream analyses. Across all samples, deamination rates exceeded 99.5%, and overall data yields were very high. The number of raw reads per sample ranged from 742,873,890 to 1,167,229,404, yielding sequencing depth between 44.93x and 70x coverage. Mapping efficiencies were high (64.1%–77.8%), indicating robust library performance and deamination efficiency across samples (Table 1). Principal component analysis based on methylated regions (see Methods for details) clearly separated adapted cells from control cells (Figure 3a), suggesting the presence of adaptation-related methylation differences. Indeed, DMR analysis detected substantial numbers of differentially methylated regions, many of which were unique for specific media adaptations (Figure 3b). DMRfinder was configured to group CpGs into regions with sizes ranging 100–500 bp, to ensure consistent region size and avoid excessively short or long segments. Next, the distribution of the identified DMRs was mapped to different genomic regions, including intergenic regions, promoter regions, and gene bodies (exons and introns) (Supplementary Table S2). This analysis identified the predominant localization of DMRs in intergenic regions, with varying but smaller contributions from promoter and gene body regions.

Lastly, we explored those genes further that were associated with DMRs and subjected them to gene ontology analysis. This revealed distinct sets of enriched pathways for the four media adaptations (Figure 3c), suggesting media-specific epigenetic reprogramming during adaptation.

**FIGURE 4:**
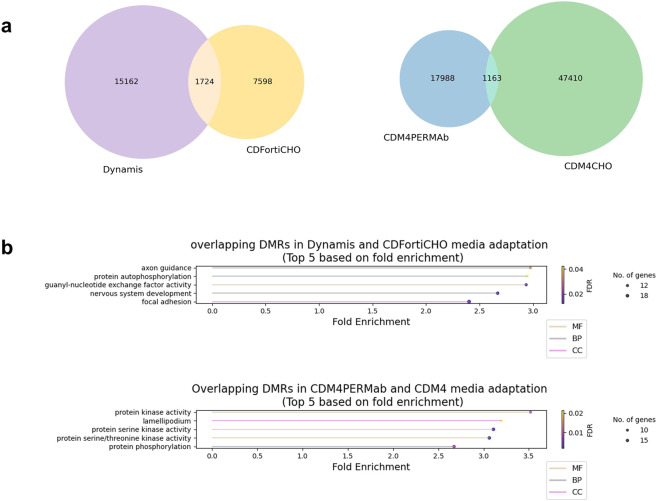
Analysis of productivity-related methylation differences. **(a)** Left panel: Venn diagram for the DMRs in Dynamis and CDFortiCHO media adaptation. Right panel: Venn diagram for the DMRs in CDM4PERMAb and CDM4CHO media adaptation. **(b)** Upper panel: top 5 GO terms ranked by fold enrichment from overlapping DMRs in Dynamis and CDForti media adaptation. Lower panel: top 5 GO terms ranked by fold enrichment from overlapping DMRs in CDM4PERMAb and CDM4CHO media adaptation. The x-axis indicates fold enrichment of each GO term. Dot size represents the number of genes annotated to each term, and dot color corresponds to the false discovery rate (FDR). GO categories are indicated by line color: Molecular Function (MF), Biological Process (BP), and Cellular Component (CC).

The differential productivities of cells adapted to Dynamis and CDFortiCHO (unchanged/decreased) and cells adapted to CDM4PERMab and CDM4CHO (increased) also provided us with an opportunity to investigate productivity-associated methylation differences. Analysis of the corresponding DNA methylation datasets identified 1,724 shared DMRs in Dynamis and CDFortiCHO media adaptation and 1,163 shared DMRs in CDM4PERMAb and CDM4CHO media adaptation, respectively (Figure 4a). Gene ontology analysis of the genes that were associated with DMRs identified distinct sets of enriched pathways for decreased and increased protein production (Figure 4b), suggesting that DNA methylation can reflect differences in protein productivity. Interestingly, these GO terms strongly overlapped with the adaptation-associated terms (Figure 3c), including focal adhesion*,* axon guidance*,* protein phosphorylation*,* and guanyl-nucleotide exchange factor activity. This overlap indicates that productivity-related methylation changes largely occur within the same functional networks that were identified in our media-adaption analyses.

**FIGURE 5:**
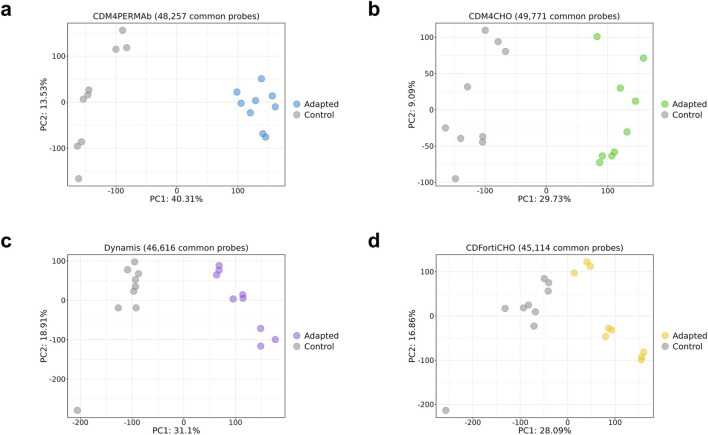
Validation of adaptation-dependent DNA methylation changes by array-based methylation analysis. Principal component analyses for matched media adaptation and control datasets, based on the beta values of all detected CpGs by the microarray are shown for **(a)** CDM4PERMAb, **(b)** CDM4CHO, **(c)** Dynamis and **(d)** CDFortiCHO media adaptation. A clear separation of adapted and control samples is seen in all cases. Note that DNA from six biological replicates was used for both whole-genome bisulfite sequencing and array-based methylation analysis. Also, DNA from three additional biological replicates of both the control and adapted groups, derived from an independent experiment, was used for array-based methylation analysis.

Finally, to validate adaptation-dependent DNA methylation changes we developed a technology platform for streamlined DNA methylation analysis. This platform was based on customized microarrays with highly specific probes that query the methylation level of n = 64,107 selected CpG dinucleotides in the CHO genome, respectively (see Methods for details). Array-based methylation profiling was carried out on the same DNA samples used for whole-genome bisulfite sequencing and on DNA samples from three additional biological replicates from an independent experiment. Principal component analysis based on the beta values of all CpGs detected by the microarray clearly separated adapted cells from control cells (Figure 5). These findings provide orthogonal validation of the results obtained by whole-genome bisulfite sequencing and establish array-based methylation profiling of CHO cells as a promising approach to optimize CHO cell culture conditions and to monitor or predict performance of the CHO cells during culturing.

## Discussion

DNA methylation is a key mechanism that allows animal cells to adapt to changing environments (Feil and Fraga, 2012; Feinberg, 2007). Understanding DNA methylation dynamics in CHO cell culture therefore has significant potential for optimizing CHO production efficiency and safety. Our study addresses an important bottleneck in the field (Kildegaard et al., 2013; Stolfa et al., 2018) by providing 57 high-quality datasets with base-resolution DNA methylation maps under different culture conditions. Indeed, CHO cells have been found to modulate their gene expression via DNA methylation in order to maintain cellular homeostasis throughout the course of a batch or fed-batch culture (Feichtinger et al., 2016; Hernandez et al., 2019; Sha et al., 2021). Moreover, marked differences in epigenetic dynamics have been observed for a single cell line over a prolonged culture time (Riedl et al., 2025). Our results support and expand these findings, as we observed significant changes in methylation patterns over the course of a fed-batch culture. Notably, these alterations correlated with the different phases of growth and were most pronounced between the lag phase and the stationary phase. This is consistent with previous findings, where methylation levels were shown to be increased during the transition from the exponential to the stationary phase, with a corresponding decrease in gene expression (Feichtinger et al., 2016; Hernandez et al., 2019; Sha et al., 2021).

Despite cell culture conditions remaining relatively constant over the course of a fed-batch culture, we detected significant changes in the methylome of CHO cells. As such, we sought to investigate methylation alterations arising from various physical and metabolic cell culture stresses known to lead to noticeable phenotypic changes. More specifically, we focused on stresses commonly observed during the course of bioprocessing, such as hyperosmolality, lactate accumulation and no glutamine availability, as these would have the most pronounced impact on the production of therapeutic proteins (Feichtinger et al., 2016; Ha et al., 2022; Nolan and Lee, 2011; Pereira et al., 2018). Although most of these single-parameter stresses caused significant changes in the protein productivity of our fed-batch cultures, WGBS analysis revealed no discernible differences in the genome-wide methylation patterns of control cells and stressed cells on the final day of culture (data not shown). As recent studies have detected changes at both the proteomic and transcriptomic levels of CHO cells in response to external stresses such as pH, osmolality, and temperature changes (De Marco et al., 2025; Desmurget et al., 2024; Riedl et al., 2025), this suggests that the stable fixation of defined DNA methylation changes over relatively short culture times might require more complex changes in the culture environment.

In contrast to single-parameter stresses, the process of media adaptation exposes CHO cells to different media compositions over the course of 2 weeks. This combination of multiple environmental stressors and extended periods of culture may better promote the long-term fixation of DNA methylation modifications used by cells to regulate their gene expression. In fact, previous research has found evidence of CHO cells modifying their DNA methylation patterns in response to adaptation to different media, with adapted cells exhibiting >10,000 DMRs compared to non-adapted cells (Feichtinger et al., 2016; Hernandez et al., 2019). Additionally, a transcriptomic analysis of adherent CHO-K1 cells that were adapted to suspension growth in protein-free medium revealed an enrichment of GO-terms linked to the extracellular matrix, response to stress, and glutathione metabolism (Shridhar et al., 2017). However, such evidence was based only on single or duplicate replicates and was limited in sequencing depth. In contrast, our findings are based on the analysis of six independent replicates, and an approx. 10-fold higher sequencing depth per replicate. Hence, our datasets provide considerably greater statistical power for downstream analyses and represent a valuable resource for further data mining.

During media adaptation three out of the four commercially available media analyzed in this study, induced marked changes in growth kinetics, antibody titre and productivity. While CDFortiCHO-adapted cells showed no significant difference in growth kinetics, DNA methylation analysis detected a substantial number of DMRs between adapted and non-adapted cells. Furthermore, a comparison of the DNA methylation patterns of Dynamis- and CDM4PERMAb-adapted cells revealed overlapping DMRs, despite contrasting changes in productivity. These results suggest that the process of adaptation itself causes changes in similar parts of the methylome, possibly reflecting a dynamic genome compartment involved in media adaptation. As the results from our Principal Component Analyses clearly suggested the presence of systematic methylation differences between the different conditions in a high number of replicates, our findings illustrate the potential of DNA methylation analysis for the establishment of epigenetic markers to monitor and facilitate the successful adaptation of CHO cells to new media.

Finally, the customized microarray platform for DNA methylation analysis in CHO cells successfully validated adaptation-dependent epigenetic changes. Our array-based findings, which again demonstrated a clear separation of adapted and control cells, provide orthogonal support for the WGBS data. Importantly, the array-based method, by focusing on a targeted set of more than 63,000 dinucleotides, offers a more streamlined and efficient approach compared to WGBS. The ability to precisely quantify methylation at dynamically modified loci suggests that the technology could be used for future CHO biomarker development to support the optimization of production processes.

In future studies, data collected from CHO cells could be used as an input for machine learning models that can utilise early-stage DNA methylation data to predict cell fates, allowing for more controlled regulation of protein productivity and more streamlined selection of desirable clones. Our novel CHO DNA methylation array could greatly facilitate these projects, as it allows for a streamlined and cost-effective analysis of large sample numbers. Nevertheless, the precise relationship between DNA methylation modifications and changes in levels of gene expression or other phenotypes in CHO cells is yet to be established (Marx et al., 2022). Hence, future work involving integrative multi-omics profiling (Kildegaard et al., 2013; Stolfa et al., 2018) is crucial in establishing a more definitive link, which would provide a basis for the development of epigenetic markers for the selection and monitoring of stable production phenotypes.

## Methods

### Cell culture

All experiments were conducted using the Chinese Hamster Ovary Humira431 cell line (A*Star BTI), which was derived from transgenic CHO DG44 cells modified to express a therapeutic antibody (Adalimumab biosimilar). For a fed-batch (14 days) or batch culture (7 days), cells were seeded at 3 × 10^5^ cells/mL in 30 mL media in 125 mL shake flasks and maintained at 37 °C, 8% CO_2_, and 150 rpm. For fed-batch cultures, 10% v/v of EX-CELL Advanced CHO Feed one without glucose (24368C, Merck) was added every other day from days 3–14, and D-(+)-Glucose solution (G8769, Merck) was added when glucose concentration dropped to 2 g/L. The cultures were monitored every day from day 3 onwards for viable cell density (VCD), cell viability, and productivity. The cell pellets were collected on day 3, 7 and 14 from the flasks for genomic DNA isolation.

### Media adaptation

Cells were passaged only when they reached a minimum of 2 × 10^6^ cells/mL and a viability of >95%. All media was supplemented with 6 mM L-glutamine (G7513, Merck) and 250 nM MTX (M8407, Sigma-Aldrich) before use. Direct adaptation was carried out for Dynamis media (A2261501, Thermo Fisher Scientific), CDFortiCHO media (A1148301, Thermo Fisher Scientific) and HyClone CDM4PERMAb media (SH30871.02, Cytiva), with the cells directly being cultured in 100% of the alternative media from EX-CELL Advanced CHO Fed-batch media and passaged at least 4 times over 2 weeks (Figure 1b). Adaptation of Humira431 cells to CDM4CHO media (SH30558.02, Cytiva) was carried out progressively with cells seeded in EX-CELL Advanced CHO Fed-batch media and CDM4CHO media in a 50:50 ratio and maintained till the cell density reached a minimum of 2 × 10^6^ cells/mL with a viability of >95%. This was followed by the second passage where the ratio of alternative media to EX-CELL Advanced CHO Fed-batch media was increased to 75:25 and the process was repeated until cells were able to maintain a viability of >97% in 100% of the alternative media (Figure 1b). The culture was then maintained in 100% new media for at least two passages for adaptation. A control culture of Humira431 cells was concurrently maintained for four passages in EX-CELL Advanced CHO Fed-batch media (Figure 1a). To compare the productivity of the cells in different adapted media, batch cultures of 7 days were performed in biological replicates, where viable cell density (VCD), cell viability, and productivity were monitored every day from day 3 of the culture (Figure 1c). The cell pellets for both control and adapted cells were collected on day 6/7 of the culture for genomic DNA isolation.

### Analytical measurements

Samples were taken from each flask daily, starting from day 3, until the end of the culture for cell count and metabolite analysis. Viable cell density (VCD) was determined using the Vi-CELL BLU analyzer (Beckman Coulter). The Cedex Bio Analyzer (Roche) was used according to the manufacturer’s instructions to measure protein titer and metabolite concentrations. Protein productivity was calculated by using the formula: Productivity (pg/cell) = Antibody Titer (pg/mL)/VCD (cells/mL). Integrated Viable Cell Density (cells·days/mL) was calculated using the trapezoidal rule as: IVCD = Σ[(VCD_(i)_ + VCD_(i+1)_)/2 × (t_(i+1)_ − t_(i)_)], where VCD is the viable cell density at each time point t. Specific productivity was calculated by using the formula: Specific productivity (pg/cell/day) = Titre (pg/mL)/Integrated Viable Cell Density (cells·days/mL). See Supplementary Table S1 for detailed results.

### Genomic DNA isolation

Cell pellets were collected from each flask on the final day of culture for DNA isolation. Cell pellets were isolated using the MagMAX DNA Multi-Sample Ultra 2.0 Kit (Applied Biosystems, A36570) on a KingFisher Apex Sample Purification System (Thermofisher Scientific) according to the protocol provided by the manufacturer. Samples were eluted in 200 µL of Nuclease-Free Water (Thermofisher Scientific, R0582). The eluted DNA was centrifuged at 20,000 g for 5 min to remove any residual beads from the isolation and quantified on the Agilent Tape Station (Agilent Technologies) to determine concentration and DNA integrity before being stored at −20 °C for further use. Genomic DNA with a DNA Integrity Number (DIN) greater than eight was used as the quality criterion for inclusion in whole-genome bisulfite sequencing.

### Whole-genome bisulfite sequencing

Samples were pre-processed using the EZ DNA Methylation-Gold™ Kit and the VAHTS Universal Pro DNA Library Prep Kit, before undergoing whole-genome bisulfite sequencing (WGBS) on an Illumina NovaSeq 6,000 platform using a paired-end sequencing protocol with read lengths of 150 base pairs. Raw WGBS data was processed using FastQC (https://www.bioinformatics.babraham.ac.uk/projects/fastqc/) for quality control and TrimGalore for trimming the adapters (https://github.com/FelixKrueger/TrimGalore). The aligned reads were mapped to the CriGri-PICRH-1.0 reference genome using Bismark (Krueger and Andrews, 2011), which was also used for deduplication and extraction of beta values for each CpG site.

### Downstream DNA methylation data analysis

CpG sites with coverage of more than 10 reads were grouped into genomic regions using DMRfinder (Gaspar and Hart, 2017), applying a minimum region length of 100bp and a maximum of 500bp to capture focal methylation changes while avoiding excessively large regions. Differential methylation analysis was performed with methylKit (Akalin et al., 2012) using logistic regression with overdispersion correction. DMRs were defined based on a minimum methylation difference threshold of 15%, a false discovery rate Benjamini–Hochberg adjusted p value <0.01, and ≥3 CpG sites per region. DMRs were considered shared between conditions when they exhibited ≥50% reciprocal overlap in genomic coordinates. Following DMR identification, associated genes were annotated using the reference GTF file based on the coordinates. DMRs were mapped to gene features such as promoters, transcripts, etc. Genes associated with DMRs were subjected to Gene Ontology (GO) enrichment analysis to identify overrepresented biological processes, molecular functions, and cellular components. Enrichment results were filtered based on adjusted p-values (FDR < 0.05).

### DNA methylation arrays

Methylation arrays were based on the Illumina Infinium methylation platform (Pidsley et al., 2016) containing more than 63,000 methylation probes specifically targeting CpG sites in the CHO genome that located to promoters and known variably methylated regions, such as LMRs (Böhl et al., 2024). The raw methylation array data were processed using SeSAMe (Zhou et al., 2018) with standard settings for custom arrays and an array-specific manifest file. Principal component analysis (PCA) was using the prcomp() function in R.

## Data Availability

Whole-genome bisulfite sequencing data has been deposited in the Sequence Read Archive (SRA) database under BioProject: PRJNA1314394.
