# Supplementary material for: Whole-genome DNA methylation analysis of Chinese hamster ovary cells undergoing media adaptation
Source: Front Bioeng Biotechnol. 2026 Jan 21;14:1716758. doi: 10.3389/fbioe.2026.1716758 (PMC12868252; doi:10.3389/fbioe.2026.1716758)
Supplement: Supplementary file 1 [file Supplementaryfile1.docx]

**Supplementary Information**

**Whole-genome DNA methylation analysis of Chinese Hamster Ovary cells undergoing media adaptation**

Suki Roy^1^, Jasrene Kaur Sandhu^1^, Lingzhi Huang^1^, Abraham Wong^1^, Guo Xuan Wan^1^, Frank Lyko^2^, Emeka Ignatius Igwe^1^, Florian Böhl^3^

^1^Evonik (SEA) Pte Ltd. Asia Research Hub. 21 Biopolis Road. Nucleos Tower A (South). Singapore 138567; ^2^Division of Epigenetics. DKFZ-ZMBH Alliance. German Cancer Research Center. 69120. Heidelberg. Germany; ^3^Creavis. Evonik Operations GmbH. Rodenbacher Chaussee 4. 63457 Hanau-Wolfgang. Germany

Corresponding author: Dr. Florian Böhl: florian.boehl@evonik.com

**Supplementary Table S1.** Additional cell culture data. The table shows Viable Cell Counts (cells/ml), Integrated Viable Cell Density (IVCD = 10⁶ cells·days/mL), titre (mg/L), productivity (pg/cell), and specific productivity (pg/cell/day) data for all samples from the experimental groups during the growth phase and media adaptation. Samples that were analyzed by WGBS are shaded in grey.

| **Experimental  group** | **Culture type** | **Sample**  **Name** | **Culture day for sampling** | **Viable cell count (x10⁶ cells/ml)** | **IVCD (x10⁶ cells·days/**  **mL)** | **Titre (mg/L)** | **Productivity (pg/cell) = Titre/Cell count** | **Specific productivity (pg/cell/day) = Titre/IVCD** |
| --- | --- | --- | --- | --- | --- | --- | --- | --- |
| **Growth Curve** | Control | HUM FB2-1 D3 | 3 | 1.61 | 2.47 | <10mg/L (too low to detect) | | |
|  |  | HUM FB2-2 D3 |  | 1.84 | 2.71 | <10mg/L (too low to detect) | | |
|  |  | HUM FB2-3 D3 |  | 1.73 | 2.33 | <10mg/L (too low to detect) | | |
|  |  | HUM FB2-1 D7 | 7 | 9.38 | 22.34 | 162 | 17.27 | 7.25 |
|  |  | HUM FB2-2 D7 |  | 9.28 | 22.71 | 169 | 18.21 | 7.44 |
|  |  | HUM FB2-3 D7 |  | 9.48 | 22.07 | 160 | 16.88 | 7.25 |
|  |  | HUM FB2-1 D14 | 14 | 18.76 | 138.67 | 1448 | 77.19 | 10.44 |
|  |  | HUM FB2-2 D14 |  | 18.74 | 132.86 | 1341 | 71.56 | 10.09 |
|  |  | HUM FB2-3 D14 |  | 15.1 | 131.46 | 1231 | 81.52 | 9.36 |
| **Dynamis adapted** | Control | HM_DYN_D7C1 | 7 | 7.65 | 24.93 | 234 | 30.59 | 9.39 |
|  |  | HM_DYN_D7C2 |  | 7.76 | 24.41 | 235 | 30.28 | 9.63 |
|  |  | HM_DYN_D7C3 |  | 7.21 | 22.86 | 238 | 33.01 | 10.41 |
|  |  | HM DYN2 D7 C1 |  | 9.96 | 40.44 | 162 | 16.27 | 4.01 |
|  |  | HM DYN2 D7 C2 |  | 11.9 | 39.21 | 163 | 13.7 | 4.16 |
|  |  | HM DYN2 D7 C3 |  | 10.1 | 40.71 | 158 | 15.64 | 3.88 |
|  |  | HM DYN3 D7 C1 |  | 14.65 | 51.09 | 218 | 14.88 | 4.27 |
|  |  | HM DYN3 D7 C2 |  | 15.35 | 50.41 | 216 | 14.07 | 4.28 |
|  |  | HM DYN3 D7 C3 |  | 15.53 | 52.27 | 209 | 13.46 | 4 |
|  | Adapted | HM_DYN_D7A1 | 7 | 10.7 | 31.8 | 118 | 11.03 | 3.71 |
|  |  | HM_DYN_D7A2 |  | 10.9 | 31.38 | 110 | 10.09 | 3.51 |
|  |  | HM_DYN_D7A3 |  | 10 | 31.16 | 117 | 11.7 | 3.75 |
|  |  | HM DYN2 D7 A1 |  | 7.56 | 28.11 | 105 | 13.89 | 3.74 |
|  |  | HM DYN2 D7 A2 |  | 7.62 | 27.94 | 103 | 13.52 | 3.69 |
|  |  | HM DYN2 D7 A3 |  | 6.69 | 28.85 | 104 | 15.55 | 3.61 |
|  |  | HM DYN3 D7 A1 |  | 11.3 | 28.62 | 101 | 8.94 | 3.53 |
|  |  | HM DYN3 D7 A2 |  | 11.3 | 31.61 | 97 | 8.58 | 3.07 |
|  |  | HM DYN3 D7 A3 |  | 11.3 | 30.99 | 98 | 8.67 | 3.16 |
| **CDM4CHO adapted** | Control | HUM CDM4 D6 C1 | 6 | 7.6 | 21.04 | 109 | 14.34 | 5.18 |
|  |  | HUM CDM4 D6 C2 |  | 7.4 | 21.1 | 109 | 14.73 | 5.17 |
|  |  | HUM CDM4 D6 C3 |  | 7.2 | 20.1 | 111 | 15.42 | 5.52 |
|  |  | CDM4b D6 C1 |  | 12.4 | 25.21 | 117 | 9.44 | 4.64 |
|  |  | CDM4b D6 C2 |  | 12.2 | 25.1 | 121 | 9.92 | 4.82 |
|  |  | CDM4b D6 C3 |  | 12.4 | 25.03 | 120 | 9.68 | 4.79 |
|  |  | HM3 CDM4 D6 C1 |  | 15.27 | 35.7 | 180 | 11.79 | 5.04 |
|  |  | HM3 CDM4 D6 C2 |  | 15.68 | 35.35 | 175 | 11.16 | 4.95 |
|  |  | HM3 CDM4 D6 C3 |  | 15.34 | 32.05 | 176 | 11.47 | 5.49 |
|  | Adapted | HUM CDM4 D6 A1 | 6 | 4.5 | 16.12 | 117 | 26 | 7.26 |
|  |  | HUM CDM4 D6 A2 |  | 4.3 | 16.58 | 112 | 26.05 | 6.76 |
|  |  | HUM CDM4 D6 A3 |  | 4.4 | 16.18 | 118 | 26.82 | 7.29 |
|  |  | CDM4b D6 A1 |  | 5.06 | 16.96 | 112 | 22.13 | 6.6 |
|  |  | CDM4b D6 A2 |  | 4.94 | 16.74 | 111 | 22.47 | 6.63 |
|  |  | CDM4b D6 A3 |  | 5.28 | 17.46 | 113 | 21.4 | 6.47 |
|  |  | HM3 CDM4 D6 A1 |  | 3.42 | 13.68 | 116 | 33.88 | 8.48 |
|  |  | HM3 CDM4 D6 A2 |  | 3.84 | 15.33 | 118 | 30.72 | 7.7 |
|  |  | HM3 CDM4 D6 A3 |  | 3.9 | 14.75 | 117 | 30.01 | 7.93 |
| **CDFortiCHO adapted** | Control | Hum-MACDF D7 C1 | 7 | 6.29 | 21.19 | 188 | 29.89 | 8.87 |
|  |  | Hum-MACDF D7 C2 |  | 5.9 | 20.24 | 189 | 32.03 | 9.34 |
|  |  | Hum-MACDF D7 C3 |  | 6.41 | 20.97 | 187 | 29.17 | 8.92 |
|  |  | HUM MA2 D7 C1 |  | 10.1 | 29.89 | 159 | 15.74 | 5.32 |
|  |  | HUM MA2 D7 C2 |  | 10 | 29.8 | 161 | 16.1 | 5.4 |
|  |  | HUM MA2 D7 C3 |  | 9.5 | 28.45 | 161 | 16.95 | 5.66 |
|  |  | MA3 D7 C1 |  | 14.65 | 51.09 | 218 | 14.88 | 4.27 |
|  |  | MA3 D7 C2 |  | 15.35 | 50.41 | 216 | 14.07 | 4.28 |
|  |  | MA3 D7 C3 |  | 15.53 | 52.27 | 209 | 13.46 | 4 |
|  | Adapted | Hum-MACDF D7 A1 | 7 | 3.24 | 8.32 | 118 | 36.42 | 14.18 |
|  |  | Hum-MACDF D7 A2 |  | 2.58 | 6.88 | 98 | 37.98 | 14.24 |
|  |  | Hum-MACDF D7 A3 |  | 3.31 | 8.59 | 115 | 34.74 | 13.39 |
|  |  | HUM CDF2 D7A1 |  | 13.4 | 38.8 | 154 | 11.49 | 3.97 |
|  |  | HUM CDF2 D7A2 |  | 13.5 | 39.3 | 158 | 11.7 | 4.02 |
|  |  | HUM CDF2 D7A3 |  | 12.7 | 39.43 | 154 | 12.13 | 3.91 |
|  |  | MA3 D7 Forti1 |  | 14.68 | 42.82 | 153 | 10.43 | 3.57 |
|  |  | MA3 D7 Forti2 |  | 14.28 | 44.08 | 148 | 10.37 | 3.36 |
|  |  | MA3 D7 Forti3 |  | 14.13 | 43.43 | 146 | 10.34 | 3.36 |
| **CDM4PERMab adapted** | Control | HM Per2 D6 C1 | 6 | 12 | 43.24 | 158 | 13.17 | 3.65 |
|  |  | HM Per2 D6 C2 |  | 11.1 | 41.65 | 157 | 14.14 | 3.77 |
|  |  | HM Per2 D6 C3 |  | 11.7 | 43.83 | 160 | 13.68 | 3.65 |
|  |  | HM PER3 D6 C1 |  | 15.2 | 40.44 | 104 | 6.84 | 2.57 |
|  |  | HM PER3 D6 C2 |  | 14.7 | 39.21 | 105 | 7.14 | 2.68 |
|  |  | HM PER3 D6 C3 |  | 14.4 | 40.71 | 107 | 7.43 | 2.63 |
|  |  | MA3 PER D6 C1 |  | 13.6 | 40.44 | 127 | 9.34 | 3.14 |
|  |  | MA3 PER D6 C2 |  | 13.8 | 39.21 | 125 | 9.06 | 3.19 |
|  |  | MA3 PER D6 C3 |  | 13.6 | 40.71 | 125 | 9.19 | 3.07 |
|  | Adapted | HM Per2 D6 A1 | 6 | 7.55 | 39.5 | 283 | 37.48 | 7.16 |
|  |  | HM Per2 D6 A2 |  | 8.25 | 41.68 | 267 | 32.36 | 6.41 |
|  |  | HM Per2 D6 A3 |  | 8.26 | 41.91 | 265 | 32.08 | 6.32 |
|  |  | HM PER3 D6 A1 |  | 13.75 | 40.78 | 229 | 16.65 | 5.62 |
|  |  | HM PER3 D6 A2 |  | 13.1 | 40.87 | 230 | 17.56 | 5.63 |
|  |  | HM PER3 D6 A3 |  | 13.32 | 42.49 | 222 | 16.67 | 5.22 |
|  |  | MA3 PER D6 A1 |  | 8.62 | 40.78 | 260 | 30.16 | 6.38 |
|  |  | MA3 PER D6 A2 |  | 8.94 | 40.87 | 253 | 28.3 | 6.19 |
|  |  | MA3 PER D6 A3 |  | 8.6 | 42.49 | 258 | 30 | 6.07 |


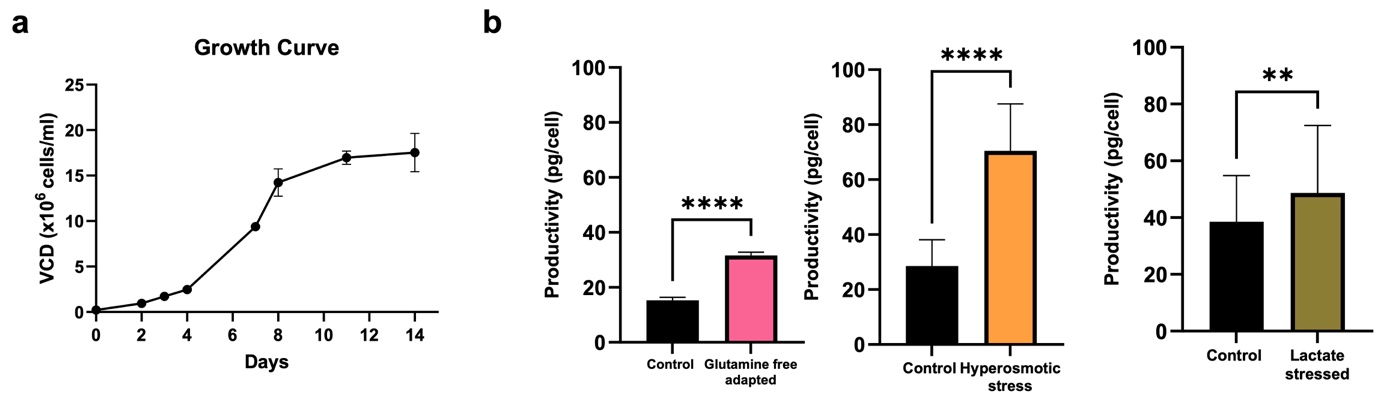


**Supplementary Figure S1. Productivity of CHO cells grown under standard fed-batch conditions vs. stress conditions.** (a) Average viable cell density of three biological replicates of Humira431 cells grown over fourteen days in a fed-batch culture. The lag phase (day 0 to day 3). exponential phase (day 4 to day 11) and stationary phase (day 11 to 14) of growth can be visually distinguished from one another. (b) Average productivities of nine biological replicates adapted to grow in glutamine-free media, hyperosmolar and lactate-stressed conditions. Asterisks indicate statistical significance: **p<0.01. ****p<0.0001 (t-test).


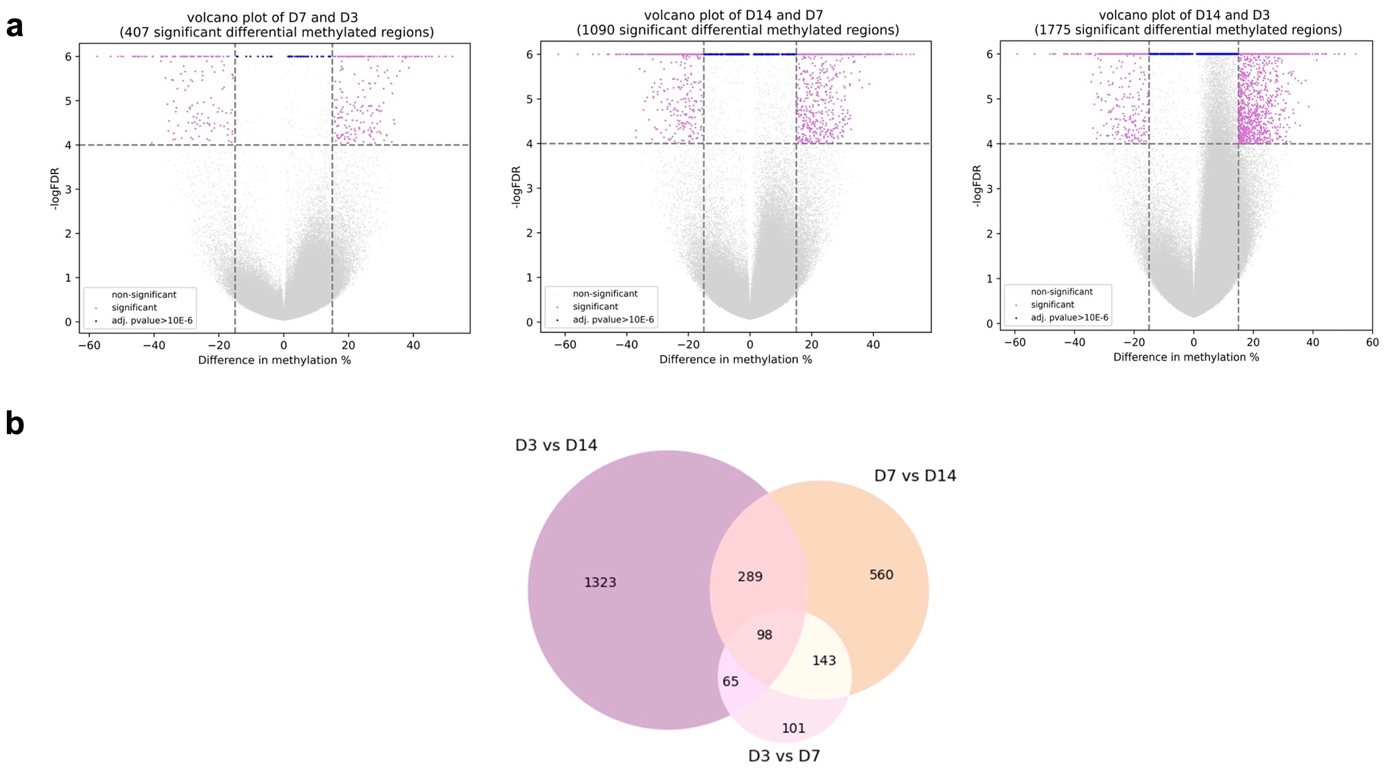


**Supplementary Figure S2. Differential DNA methylation analysis across different growth phases of a fed-batch culture.** (a) Volcano plots showing differentially methylated regions (DMRs) in the different growth phases of a 14-day CHO fed-batch culture. (b) Venn diagram showing the number of DMRs overlapping and those restricted to the specific phase of culturing.

**Supplementary Table S2.** The distribution of identified DMRs in different genomic regions. The table shows the absolute numbers and percentages of the genomic distribution of the identified Differentially Methylated Regions (DMRs).

| Experimental group (Media or overlap between media) | Absolute number DMRs | DMRs located within intergenic regions | | DMRs located in promoter regions | | DMRs located within gene bodies  (exons & introns) | |
| --- | --- | --- | --- | --- | --- | --- | --- |
|  |  | Absolute number | Percentage | Absolute  number | Percentage | Absolute  number | Percentage |
| CDM4PERMAb | 19151 | 15674 | 81.8% | 192 | 1.0% | 3285 | 17.2% |
| CDM4CHO | 48573 | 31470 | 64.8% | 555 | 1.1% | 16548 | 34.1% |
| Dynamis | 16886 | 13012 | 77.1% | 275 | 1.6% | 3599 | 21.3% |
| CDFortiCHO | 9322 | 6674 | 71.6% | 175 | 1.9% | 2473 | 26.5% |
| Overlap between CDM4PERMAb and CDM4CHO | 1163 | 897 | 77.1% | 18 | 1.5% | 248 | 21.3% |
| Overlap between Dynamis and CDFortiCHO | 1724 | 1282 | 74.4% | 22 | 1.3% | 420 | 24.4% |
